# Secular, Spiritual, and Religious Existential Concerns of Women with Ovarian Cancer during Final Diagnostics and Start of Treatment

**DOI:** 10.1155/2013/765419

**Published:** 2013-10-31

**Authors:** Lene Seibaek, Lise Hounsgaard, Niels Christian Hvidt

**Affiliations:** ^1^Department of Obstetrics and Gynaecology, Aarhus University Hospital, Brendstrupgaardsvej 100, 8200 Aarhus N, Denmark; ^2^Research Unit of Nursing, Faculty of Health Sciences, University of Southern Denmark, Campusvej 55, 5230 Odense M, Denmark; ^3^Institute of Nursing and Health Sciences, University of Greenland, Greenland; ^4^Klinik und Poliklinik für Palliativmedizin, LMU, Marchioninistr. 15, 81377 München, Germany; ^5^Research Unit of Health, Man and Society (50%), Institute of Public Health, SDU, Odense J. B. Winsløwsvej 9B, 5000 Odense C, Denmark; ^6^Freiburg Institute for Advanced Studies (FRIAS), Freiburg University, Albertstraße 19, 79014 Freiburg, Germany

## Abstract

*Introduction*. This paper deals with secular, spiritual, and religious existential concerns during severe illness. *Materials and Methods*. Qualitative research interviews were made before and after surgery with women who underwent final diagnostics, surgery, and chemotherapy for ovarian cancer. By applying a phenomenological-hermeneutic text interpretation methodology the findings were systematically identified, placed into meaning structures, interpreted, and critically discussed. *Results*. The analysis offered insight into the complexity of challenges and personal development over time in being a woman with ovarian cancer during her first treatment period. Although the women experienced their health to be seriously threatened, they also felt hope, will, and courage. The diagnostic procedures and treatment had comprehensive impact on their lives. However, hope and spirituality were important resources of comfort and meaning. *Conclusion*. Hope and courage to face life represent significant personal resources that are created not only in the interplay between body and mind but also between patients and their healthcare professionals. The women dealt with this in a dialectical manner, so that hope and despair could be present simultaneously. In this process secular, spiritual, and religious existential meaning orientations assisted the women in creating new narratives and obtain new orientations in life.

## 1. Introduction

A vast body of international research literature points towards a consistent link between hope, spirituality, and health and describes the way spiritual and religious resources influence the physical and psychosocial well-being [1]. Based on the existing literature la Cour and Hvidt have concluded that when struggling with crisis and despair, humans variously employ secular, spiritual, and religious existential meaning resources [2]. This has also applied to the findings presented in this paper, even though our study was conducted in Denmark, what is known to be “the least religious nation in the world.”

In our previous research of the lived experiences of Danish women undergoing ovarian cancer surgery we found that the women, despite being in a most vulnerable situation, demonstrated substantial inner resources in terms of hope, strength, will, and courage to face life [3, 4]. As they perceived inducing and strengthening hope to be very important to their survival, they put a strong emphasis on having a positive versus a negative mindset. Remarkably, this did not seem to contrast being fully aware of the risk of a fatal outcome.

While the concept of hope is by no means clearly defined, unifying or narrowing that concept is not the intention of this paper. In a concept analysis based on a literature review, Benzein and Saveman identified 54 different definitions of hope [5]. Nevertheless, the authors argued for further widening of the understanding of the concept. We agree that limiting the definition might lead to the exclusion of important, not yet fully investigated aspects of hope. As hope was often studied in relation to burdensome or negative life events, Benzein and colleagues stressed, with reference to the work of the French philosopher Gabriel Marcel (1889–1973), that hope is experienced—and can be studied—where the temptation to despair exists [5, 6]. When people live their lives free of any major threat, they live in the general state of hoping that their lives will continue in the way in which they are accustomed. When threatened, however, this general state of hope mutates into an active state of hoping [7]. We find our work in line with this understanding. The diagnosis and treatment of a serious cancer disease has proven to be a period of time in which hope—like despair—is very much present. 

## 2. Materials and Methods

### 2.1. Context

With only 2% of the population going to church once a week, Denmark represents a country where organized religion is hardly practised at all. 

The study took place in a Danish University Hospital at a regional centre for surgical treatment of gynaecological cancer diseases. Among women suffering from gynaecological malignancies in the western world, ovarian cancer is the leading cause of death. Danish women have a very high incidence of and mortality rate from this disease [8]. During the last decade the five-year survival has been stable at approximately 40 percent [9]. This is probably because the majority of the women (66%) are being diagnosed in the advanced FIGO stages III and IV (FIGO: International Federation of Gynaecology and Obstetrics) [10]. In addition, 25% suffer from various sorts of comorbidity, a situation that seriously impacts health care seeking, the ability to go through effective treatment, and consequently also survival [11]. The aetiology of ovarian cancer is mostly unknown, but approximately 10% of patients have a hereditary component [12]. Furthermore, some correlation between lifestyle in terms of oral hormone therapy, a high intake of milk products, smoking, and excessive weight and the development of cancer or borderline tumours in the ovaries has been demonstrated [13]. The initial symptoms are often vague and nondisease specific, and there are no valid screening methods available [14]. The clinical pathway consists of diagnostic procedures, surgery and care, microscopy of the removed tissue, and followup, all within a period of three to four weeks [15]. Due to the nature of the disease, it is often not possible to obtain a verified diagnosis prior to the surgery. At the start of treatment this circumstance puts major psychological strains on the women as well as on their families [3]. After the surgery, the clinical pathway depends on the final diagnosis and staging. Women with spread of the disease receive chemotherapy in a series of four to six treatments. Women with localised or borderline disease are treated with surgery alone and scheduled for regular followups. Women with benign conditions have completed their treatment after the initial surgery. 

### 2.2. Selection of Participants

Based on the results from the registry study concerning living conditions, age, and diagnoses, the women were strategically selected one by one via medical records to represent typical variations in the population of Danish ovarian cancer patients [16]. The study inclusion procedure was carried out when the women arrived at the hospital the day before surgery (Table 1). Women who were mentally ill, or did not speak Danish, were not selected. 

### 2.3. Generation of Data

To gain insight into personal experiences from an individual life-world perspective, semistructured qualitative research interviews were used as the data collection method [17]. The experienced world, which gradually emerged through the interviews, was an interpreted world. Therefore, the interviews were analysed within phenomenological-hermeneutic interpretation theory inspired by Ricoeur, in which phenomenological description and hermeneutic interpretation are combined with the aim of explaining better in order to understand more [18, 19]. The interviews were performed with each woman alone with one interviewer. They were conducted by the first author, who has a professional background as a specialist nurse in cancer care. The interviews followed semistructured guides (Table 2). Further questions were based on the participants' individual reflections and experiences, with each woman being interviewed twice. The first interview took place on the hospital ward at the evening before the surgery. Based on clinical experience, this period of time represented a moment of quiet reflection for many. To obtain a balanced interview setting and to gain further insight into developing experiences, a second interview was made in the participants' private homes approximately eight weeks after discharge. In this way the women were offered an opportunity to reflect on the content of the first interview and to elaborate on personal viewpoints, and the findings from those follow-up interviews reflected narrations of human experience over time [17]. In doing so, we drew on Ricoeur's concept of emplotment [20], by which the human capacity of creating new narratives of their lives is perceived to be central in order to maintain a coherent, fluid, and stable identity during times of change. The interviews were digitally recorded and transcribed verbatim. The qualitative research software NVivo8 was used to systematise the findings and prepare the text for analysis [21]. Quotations were translated from idiomatic Danish to English by a professional copy editor who was not otherwise involved in the study.

### 2.4. Data Analysis

The analyses were inspired by phenomenological-hermeneutic text interpretation theory [18], which during the last decade has been methodologically developed further within the caring sciences [22–25]. Following this methodology, the interview findings were systematically identified, put into meaning structures, interpreted, and discussed. Further analyses took place in a dialectic movement between three analytic levels: Naïve understanding, structural analysis, and critical interpretation [18]. In this way the analyses moved towards deeper understanding of what the text referred to in the world [22, 24, 26]. Initial overview and interconnected understanding of experiences and actions were developed through nonjudgemental readings and rereadings of the interviews. In order to attain valid knowledge, the text must be objectified [25]. This took place during the transcription of audio files, condensation of meaning, and a structuring of quotations into patterns and subthemes. The structural analysis moved between the parts and the whole, between the empirical findings (“what the text said”) and the meaning condensation (“what the text spoke of”) [26]. In this way the structural analysis offered an explanatory perspective of experiences and acts. In-depth analyses were made by moving back and forth between the empirical level and the analytical level, between comprehending and explaining the matter of the text. Subsequently, the findings were critically interpreted and discussed. Through the critical interpretation and discussion, the findings moved beyond an individual, subjective level towards a universal, general level, moving from being the interviewed women's personal experiences and reflections towards identifying significant, valid facets of being-in-the-world as that woman undergoes final diagnosis and ovarian cancer surgery, starts chemotherapy treatment, or finishes her sick leave [3, 15]. 

### 2.5. Ethical Considerations

As the study addressed women who were undergoing surgery for a potentially life-threatening disease, carrying it out held substantial ethical considerations. During the interview period, the interviewees were seen and met as individual persons with whom the first author, via the interviews and other kinds of interaction, had a limited but mutual relationship. The second interview took place in the interviewees' private homes. By doing so, the interviewer participated in defined areas of women's private lives. The study focus, however, remained on their personal experiences and reflections concerning the research topics. As the first author was a guest in a private home, the interaction included telephone calls, saying hello to family members and pets, drinking coffee, and engaging in other kinds of friendly conversation. 

The study was approved by the Danish Data Protection Agency (file no. 2007-41-1640). Audio files and transcripts were stored in accordance with the Agency's rules. Under the rules of the Central Denmark Region's Committees on Biomedical Research Ethics, the study did not need further approvals. The women were not invited to participate until a personal treatment plan was ready. Pursuant to the rules of the Helsinki Declaration, the women received both oral and written information before giving their consent to participate [27]. In case the interview should initiate specific worries concerning the ovarian cancer disease or the treatment, an extra follow-up visit in the outpatient clinic was offered. 

### 2.6. Strengths and Weaknesses in relation to the Applied Methodology

Conducting repeated semistructured research interviews with strategically selected participants proved sufficient to obtain rich and saturated interview data. Although it might be considered a weakness that we did not include the perspective of relatives, that design enabled the women to fully express personal concerns in relation to their family and friends. All the women, who were able to participate in both interviews, indicated that the first interview was conducted in an appropriate manner, and furthermore, the second interview enabled us to study the development of hope and the courage to face life during the perioperative period. 

The fact that the first author had been employed as specialist nurse in the study setting might have represented a limitation, as she could be influenced by preexisting perceptions and positions. On the other hand, her skills as an experienced cancer nurse and her background knowledge of the clinical aspects of the participants' medical history enabled her to stay compassionate yet still focused during the data collection. That fact that none of the interviewed women made use of the optional follow-up visit, which was offered in relation to the interviews, supports this assumption. Furthermore, intellectual distance in the subsequent analyses was sustained by awareness of potential preconceptions and by the applied interpretation methodology [26, 28]. 

## 3. Results

All the invited women agreed to participate in the interviews; however, one woman was only interviewed preoperatively due to her death shortly after the operation. The interview material finished with ten participants. At that point the data had ensured a sufficient spectrum of variances and could still be analysed as a whole [29, 30]. The interviews lasted from 19 to 111 minutes each (mean: 43 minutes). The women's average age was 58.8 years. Five women had retired, and four were living alone. At the end of the study period participants 1, 3, 6, 7, 9, and 10 had started chemotherapy because their disease had spread (Table 1). 

The naïve (nonjudgmental) reading revealed that the diagnostic procedures, surgery, and chemotherapy had comprehensive impact, not exclusively on the women's physical bodies but just as much on their everyday lives. Throughout the perioperative period, well-being and discomfort seemed to occur in cycles. As well-being and discomfort were individual experiences caused not only by the disease itself but also by its treatment, the women did not go through identical cycles. Women who suffered from late stage disease in which the ovarian cancer had spread seemed to experience overall relief of their discomfort after the surgery and during the chemotherapy. Women with localised or borderline disease who did not have any substantial physical discomfort initiating their diagnosis experienced their health to be worsened during treatment. As individual and diverse as their disease reactions were, their needs and experiences of hope were diverse and individualized.

The structural analysis established a main theme concerning “Hope and existential concerns during final diagnoses and first treatment period” (Table 3). This theme offered insight into the complexity of the challenges and personal development over time of being a woman with ovarian cancer during her perioperative period. It was based on the overall finding that although the women did experience their lives to be seriously threatened during diagnoses and start of treatment, they also felt a surprisingly strong hope, will, and courage to face life. The theme “Hope and existential concerns during final diagnoses and first treatment period” held three subthemes: “Courage to face life,” “Hope,” and “Existential concerns.” These subthemes were empirically identified through patterns in the text, which consisted of descriptions of personal intentions, actions and living conditions, personal reflections, and experiences during the study period. In the following section the subthemes will be critically interpreted and discussed as separate analytic categories. This distinction is made only for reasons of transparency and presentation, as the themes are in fact related to and mutually influenced by each other (Table 3).

## 4. Critical Interpretation and Discussion

The distribution in age, the socioeconomic status, and the living conditions of the interviewed women reflects a population where the majority are elderly, retired, and living alone with limited financial resources [16] (Table 1). Seen as a group, the Danish ovarian cancer patients can therefore be characterised as physically and socially vulnerable. 

### 4.1. Subtheme I: Courage to Face Life

Subtheme one deals with the significance of having courage to face life, and the way flexible and person-centred care could support this courage.

#### 4.1.1. Courage to Face Life Is Important, but It Is Not a Cure

Some women put a strong emphasis on having personal courage to face their present circumstances in life: “*Everybody has to face some adversity in life, right? But it's probably also my nature. When I meet a problem I simply try to solve it—I never give up!*” (6).

Some epidemiological studies have shown that courage to face life might have a certain positive impact on survival odds [31]. However, we believe one should interpret such results carefully due to the methodological challenges of investigating the phenomenon [32]. In our study courage to face life was rather seen as an approach towards life, a cradle gift, or a coping strategy which assisted those who possessed it to go through treatment the best possible way. None of the women believed that this capacity could actually fight their disease (Table 3). In a caring science perspective, courage to face life embraces concepts of strength and will [33]. *Strength* relates to the experience that the body and its reactions are well-known and predictable when healthy. During illness, the body and its reactions can change into unknown and unpredictable bodily sensations. As diagnostic difficulties are common in ovarian cancer, feelings of uncertainty and of having been let down by the body may be present. *Will *dictates that a certain amount of mind power or endurance is needed to undergo the lengthy diagnostic procedures and treatment modalities.

Some of the women reached normal levels in their physical health status during the study period; however, mental recovery seemed to be a lengthier process. This also applied to those who had their cancer diagnosis refuted or who were cured by surgery. This phenomenon is well-known in relation to survivorship, although Price et al. have found that higher symptom burden and stage of disease significantly predict posttreatment depression and anxiety [34]. Mental health and quality of life in women with ovarian cancer have been the subjects of several questionnaire studies [35, 36], but so far these have not led to ways of sufficiently addressing the patients' personal needs. Although a supportive rehabilitation program should include more extensive counseling and followup, there is evidence that telephone followups can provide some psychosocial support [37]. 

Difficulties concerning communication and approach in the transition between treatment modalities and settings were found. This reflects a well-known yet still insufficiently addressed phenomenon in the organisation of healthcare [38]. Some women experienced that, while the surgeon oncologist had focused on what was removed of the tumour, the medical oncologist focused on what was left behind. In a strictly biomedical perspective, it is obvious that such changes in focus have to do with various steps of the clinical pathway. But from a patient perspective, these changes occurred in very vulnerable phase: during transition from surgery to chemotherapy, moving from one staff to another, and from one hospital department to another. This seemed to cause the women substantial mental strain and disturbance. Considerable professional awareness of the power relations and imbalances, embedded in the organisation of healthcare, is therefore required.

#### 4.1.2. Care Can Influence the Patients' Courage to Face Life

When treatment and care were delivered in a person-centred approach, the women felt safe and cared for, not only as patients but also as persons: *“He is such a nice man (the surgeon). His wife was born in the village I come from—I knew her when she was a girl. And he said to me: I'm married to that little girl now”* (3).

As described by Travelbee, establishing a human-to human relationship [39] includes perceiving, thinking, feeling, and acting in relation to the other person. In this way, the patient can be responded to as a unique human being representing so much more than her disease. However, this requires that the healthcare professionals possess the empathy, the courage, and the willingness to breach barriers of title, position, and status. 

#### 4.1.3. To Summarize

From a patient perspective, sustaining courage to face life is crucial in going through ovarian cancer treatment. However, this courage can be put under pressure, not only by the cancer diagnosis and treatment but also by the organisation of patient pathways and by healthcare professionals' attitudes. This seems to constitute a substantial challenge especially in relation to information, transition, and approach. Some of these can however be remedied when care is delivered in a person-centred approach.

### 4.2. Subtheme II: Hope

Subtheme two deals with the way in which care influences hope, how notions of the future were revised, how personal experiences might impact hope, and with the perception that life had forever changed. The structure of hope is very complex. Most of us are not aware that we constantly live in a state of hope. Gabriel Marcel, the great French philosopher of hope, has said that: “Hope is for our soul what breathing is for our organic bodies” [6, page 9]. Our living in hope is so deeply integrated that we live in confidence that tomorrow will bring a new day. So, according to Marcel, we are normally living in the state of “universal hope” [6]. If universal hope is challenged, for instance, by a serious diagnosis, the structure of hope changes towards specific hope, hope for something, for instance, the hope for getting cured. In this way hope and hopelessness are two sides of the same coin as we grapple to come to terms with both disease threats and the prospects for being cured. 

#### 4.2.1. Care Influences Hope

Inducing and strengthening hope seemed so important that the women became reluctant to listen to healthcare professionals who indicated that being fully cured might not be an option: *“If you're not allowed to believe in the positive signals from your own body—if it's almost as if somebody tells you its wrong to believe in them—that's really not good. Because that's where hope should be and optimism too, right?”* (7).

The women dealt with this in a dialectic approach. They also expressed awareness of their generally bad prognosis and risk of a fatal outcome. However, at this point, their main focus was on staying alive, and it seemed important to them that their healthcare professionals, despite the prognosis, maintained a personal engagement and sustained hope. This was done while being realistic, as the women's active hope represented neither escape from reality nor denial.

Independent of stage and prognosis, it was not simply that their bodies were impacted by disease and treatment, it was the women's whole lives. This overall finding highlights the importance of initiating and maintaining a personalised and holistic approach right from the commencement of treatment. But specialization in healthcare does not facilitate a comprehensive approach. Considering the increasing comorbidity and the extensive treatment regimes, together with the documented positive effect of a holistic and caring approach, one might consider expanding the concept of serious disease to encompass also its impact on peoples' everyday lives [40].

In our previous research we found that hope seemed strongly related to bodies, as hope was initiated and strengthened by improvements in the physical condition [3]. Although Benzein et al. have already suggested that physical well-being might contribute substantially to the maintenance of hope [41], hope has primarily been studied and dealt with in an existential context within the caring sciences [7, 42]. It is therefore remarkable that hope and courage to face life were reinforced through fulfilment of fundamental physical needs. Although hope is related to the dependence and care of others, it may also be understood as future-oriented, specific, and active [6, 39]. 

#### 4.2.2. Notions of the Future Are Being Revised

During the study period the women revised their notions of the future more than once: “*When you get such a disease, then suddenly there's a frosted door on your time line, suddenly you cannot see that far*” (9).

Prior to their surgery, this was primarily due to fear of not surviving surgery or anaesthesia or of developing complications. The women described themselves as well informed; nonetheless they had difficulties in describing their near future in words. This situation changed from the first to the second interview, during which most women had regained the capability of making plans for the immediate future. Women who were cured by surgery dealt with long-term worries concerning recurrence and late effects, while women with late-stage disease seemed to have a much shorter time horizon. 

#### 4.2.3. Personal Experiences Can Impact Hope

When experiencing physical and mental comfort, strong feelings of hope arose. It seemed as if the positive experiences of being well-taken care of enhanced confidence and safety. *“Then he (the doctor) told me: you will never be fully cured. But I did not ask him—I know that when it comes to cancer you should never ask a doctor such a question… I think it was cruel of him to take away my hope, do not you think so too?”* (8).

Similarly, however, negative experiences enhanced feelings of insecurity and distrust. Still, these experiences also increased awareness of personal needs and expectations. As Travelbee, drawing on the work of Lynch, phrases it: “Hope can be the sense of the possible” [39, 43]. As their hope for something was triggered, previous experiences with illness and other kinds of adversity in life assisted the women in going through their present challenges. 

Substantial variation in the personal approach towards the perioperative period was found. As illustrated by interviewee three, expressing her wishes of “*getting better and get on my feet again”* (3), some did not expect themselves to be able to influence their situation on any practical level. In her point of view, she could only hope that her situation would improve: *“I hope it will happen*” (3). In a religious context, the quotation might be understood as a direct or indirect expression of prayer. Rabbi Spicvak has been quoted as saying: “*Wishing is praying, and who, if sick, does not wish with all the intensity of his soul for recovery?”* [44]. Some women were more inclined to pray to help the doctors make them well, something that has been observed both in the USA [45] and in the Nordic countries [46]. Others would not seek the influence of any higher power, relying primarily on personal strength and spirit: “*I am the one who has to go through it”* (6). In this approach, the relatives and staff were seen as the primary partners in a mutual effort.

Our attention was drawn towards the fact that only one woman represented the self-managing and resourceful patient. Compared to the physical condition, the socioeconomic and psychosocial conditions are often given less attention in healthcare [47, 48]. However, in a life with massive social problems and complex disease, an operation due to suspected ovarian cancer might merely be parenthetical. This was illustrated by interviewee five, who took care of her demented and very mobile husband 24 hours a day in a countryside cottage with no modern conveniences. Asked of her concerns regarding a potential cancer diagnosis she declared: “*I haven't done any speculations on that—I simply haven't got the time”* (5). 

Knowing or hearing of persons who had survived a serious cancer episode seemed to enable the women to create inner pictures of a life “after treatment.” In this way, narrations of survival sustained hope. This could be expanded to comprise the hope of a future life that was even better than the present: A life without stress as a happier, more vivid person; a life in which hope was simply the basis—*hope in itself*. 

#### 4.2.4. Life Has Forever Changed

The experience of a serious cancer diagnosis and the fear of recurrence represented a lifelong companion: *“The day I was told that I had a really serious cancer disease was an absolute landmark”* (9).

Although the women considered their surgical procedure to be dangerous or even life-threatening, they did not question its necessity, presumably because they perceived their condition to be irreversibly progressive or because it already felt impossible to live with. In this way the diagnosis represented a point of no return.

#### 4.2.5. To Summarize

During the study period the disease and treatment had substantial impact on the women's everyday lives. This circumstance gave rise to revised notions of their future and they began to create new narratives of their lives. Experiencing comfort and strength reinforced hope and courage to face life during this process. Hope was dealt with in a dialectic approach, from constituting a basis in life (hope itself) towards actively hoping for something. In this process hope and despair can be simultaneously present. 

### 4.3. Subtheme III: Existential Considerations

Subtheme III (Table 3) dealt with existential considerations of death, of hope being integrated into universal life, and of the way such considerations added meaning to the disease. 

#### 4.3.1. Death Becomes a Reality

The women perceived death to be the immediate consequence of developing complications or the long-term outcome of the chemotherapy not being effective: *“It just crossed my mind the other day: you could actually die during the surgery. Life is fragile.”* (7).

Talking of death in a country like Denmark, previously discussed as perhaps the least religious nation in the world, has become ever more problematic as religious discourse fades [49, 50]. But although there might be this tendency of shying away from discussing death in the population, the women spontaneously touched upon the subject the evening before their surgery. The diagnosis gave death a sudden reality through which a need to speak of their death emerged. As some of the women did not have a previously developed language for such considerations, various ways of talking around the subject were applied: *“All my folks have died of cancer. Daddy died of it—he was gone after three weeks, and Mama died of a brain tumour…. So, you think it might be something like that—but then again—it might not be that bad after all”* (3). Besides constituting a severe strain, the increased awareness of their mortality seemed to create a strong focus on staying alive. Women receiving chemotherapy continued to reflect on this: *“I've always been extremely happy about my job. But if my time horizon becomes very short, then I won't spend the rest of my life working, that's for sure. Then I will focus on my family”* (10). Even though death was not perceived as imminent, but rather something that might happen in future if chemotherapy did not have the desired effect, their reflections had become increasingly explicit and specific. 

#### 4.3.2. Personal Hope Can Be Integrated in a Universal Understanding of Life

The women described hope as multidimensional and constantly changing: “*Somehow it all makes sense, right? The universe wouldn't be the same without me—at least I think. I should be here, too. Nobody knows for how long*” (8).

The women expressed a multidimensionality of hope [51, 52]: hope for a cure, hope for a good life, long or short and in spite of disease, hope for death with dignity, hope that there would be something good waiting for them after death, and so forth. Universal hope for progress and love between human beings in a future of which they were no longer part was also expressed. Sometimes the dimensions of hope seemed to conflict, analogous to what the Canadian philosopher Charles Taylor phrases as “cross-pressures” [53, 54]. *“It was relatively easy when I was a child*—*to imagine God sitting up in the sky. The universe was so much smaller. Jesus was just nearby*—*I mean, literally”* (7). The need for meaning and something to hope for seemed so significant that some of the narratives of hope appeared to be contradictory: *“Whatever happens afterwards*—*if there is something afterwards, I'm not sure of that. I think there might be a dimension in life of what was mine. Some sort of gratitude of life*—*or the opposite. Some sort of love*—*or the opposite”* (7). As a consequence new narratives emerged: women who did not consider themselves religious, and who were otherwise informed by scientific world views, seemed to give in to a need for meaning, hoping that life would go on in some form even if their earthly death would be the immediate outcome. 

Like the notion of hope, the concepts of spirituality and religiosity are somewhat unclear. Spirituality used to be an integral part of the overarching concept of religion and religiosity and was mainly seen as lived religious life or simply piety [55]. For example, William James entitled his seminal work on the psychology of religion “The Variety of Religious Experience,” whereas today he might simply have called it the “The Variety of Spiritualities.” The term Spirituality emerged in popularity at the beginning of the 20th century during which the concept came to be used as a new word for the mystical life and more generally the life of faith or simply religious life and experience. To study the life of faith is obviously not a new invention, although spirituality was formerly embedded in and seen as an aspect of religion, never contrasted with it. However, things started to change in the beginning of the 20th century, where the concept of spirituality, disentangled from religiosity, emerged in the Alcoholics Anonymous (AA) movement with its emphasis that all attending the AA meetings should have some kind of faith, spirituality, without determining what kind of religious content and practice such spirituality should have [56]. 

Spirituality and religiosity have become differently evaluated, and Zinnbaue et al. speak of “Good spirituality, bad religion,” spirituality being defined as the broad, individualistic concept that every human can fill with her or his own meaning, whereas religiosity relates to dogmas, institutions, rituals, and communal life [57]. In relation to healthcare Koenig argues that spirituality is the best clinical precept because it is so broad that it can engage all patients; however, as a research concept it seems to be too broad and imprecise [58]. 

Although Denmark is considered as one of the least religious nations in the world, many Danes still call themselves believers without wanting to define precisely what this entails: “I'm a believer, but I'll be damned if I'm religious” as Ina Rosen entitled her PhD based on interviews with young Danes and Swedes [59]. In such a secular culture the broader and fuzzier concept of spirituality therefore seems to have more traction than the more traditional and dogmatic concept of religiosity. Along these lines it is of little surprise that the informants in this study were very careful in the way they spoke about their disease-related spiritual thoughts. Nevertheless, the findings lend insight into how Danish women with ovarian cancer may experience and describe spiritual concerns in relation to their disease. 

### 4.4. Existential Considerations Can Add Meaning to the Disease

A consistent characteristic of the existential considerations was the search for meaning—not simply the meaning of getting the disease, but just as much the meaning of life: *“I wouldn't characterise faith as being positive in itself… but it provides a sense of being here, as a human being, with a certain destiny and certain experiences”* (7).

**Figure 1:**
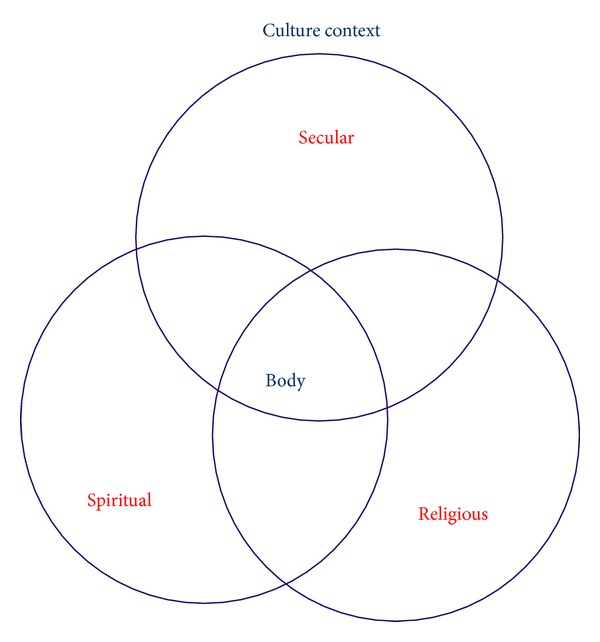
Relation of existential meaning-making domains.

Diverse meaning orientations and constructs could be organized according to the three basic dimensions of *existential meaning orientations* as delineated by la Cour and Hvidt: secular, spiritual, and religious orientations [2]. These dimensions can be clearly distinguished but are nevertheless related (Figure 1). Secular existential orientations have to do with everyday life without taking on a spiritual or religious character, and spiritual existential orientations relate to an individual spiritual life, whereas religious existential orientations are linked up with doctrines and promises of salvation and support from religious communities. Secular resources that initiated the sense of hope and meaning could, for instance, be the importance of a job, the joy of a pet animal, or the pleasures of gardening. When thinking of spiritual resources, the women would describe more-or-less conceptualized spiritual practices such as meditating or praying in moments of distress or going into nature to obtain peace in some contemplative manner. In relation to religious resources, the women might pray that they would go safely through the operation or seek comfort in thoughts of an afterlife. This was expressed in a nonreligious everyday language or as the perception of a guardian angel but could also be rooted in a specific religious community (Table 3). These quotations represent glimpses of a reality typical for secular culture. The language for existential meaning orientations is anything but bold, held in a rather nontraditional religious language, but with openness to a transcendent reality.

Some women were convinced that their disease had developed by chance and without any reason: *“I think it strikes at random”* (6), while others tended to believe that their disease held psychobiological elements such as upholding a lifestyle out of sync with nature: *“There are a far too high contents of hormones in conventional milk. And when you've got a hormone sensitive cancer disease… the farmers are pushing the production of milk too far”* (10). One woman had a heredity component in her disease development: “*I have a gene mutation that gives me a fairly large risk of getting one or the other *(breast or ovarian cancer),* so it was sort of like, I had resigned myself: It had to come sometime”* (9). This knowledge had prepared her for a potential cancer diagnosis, but on the other hand it had brought about worries and feelings of guilt: *“It has really been my biggest concern—if I pass it on to my children and grandchildren.”* (9).

#### 4.4.1. To Summarize

During final diagnoses and start of treatment, death becomes a reality. As death can be caused not only by the disease but also by its treatment, it becomes closely related to both. This generates a need to speak of death and of the meaning of life in an explicit and specific manner, which might be in conflict with previous narratives of hope and existential considerations. Through the process of existential meaning-making, new narratives and orientations can emerge.

## 5. Conclusions

To involve patient resources has become a mantra in health care, including within the context of cancer treatment and care. However, while this approach might sound obviously correct, implementing it in daily clinical practice has proven to be difficult. 

Hope and courage to face life represent significant personal resources that are created not only in the interplay between body and mind but also between patients and their healthcare professionals. The overall finding that it was not simply the women's physical bodies but rather their whole lives that became impacted by the disease and treatment highlighted the importance of maintaining a professional engagement and holistic approach from the beginning of treatment—in particular during highly specialised fast-track clinical regimes.

The women dealt with their hope dialectically so that hope and despair could be presented simultaneously. Experiencing personal comfort and strength can reinforce hope, and we find it fair to conclude that the patients' inner resources thus can be activated and strengthened by adjusted information of the disease and its treatment, psychosocial support, and physical care right from the commencement of the treatment modalities.

During final diagnosis, death is perceived to be closely related to both the disease and its treatment. Through this recognition, a need to speak of death and the meaning of life in an explicit and specific manner emerges; this need can be in conflict with the existing narratives of hope. However, existential meaning-making might assist the women in creating new narratives and through this process obtain new orientations of their lives.

## 6. Implications for Clinical Practise

Based on the findings presented in this paper we suggest the following.

- That person-centred care is trained and implemented in oncology settings.
- That healthcare professionals focus also on the general health and everyday lives of the patients during treatment.
- That physical comfort and well-being are seen, not as excessive luxury but as important tools to sustain and strengthen hope and courage to face life during treatment.
- That communication and cooperation during transitions are further developed and prioritized.
- That supportive followup and rehabilitation are offered as an integrated part of the treatment for patients in need of it.
- That patients are given the opportunity to develop, share, and adjust their narratives of illness during their treatment trajectory.

## Figures and Tables

**Table 1:** List of interview participants.

Number	Age	Diagnosis	FIGO^1^ stage	Civil status	Number of children	Socioeconomic status
1	51	Ovarian cancer	IIIC	Single	0	Employee
2	29	Ovarian cancer	I A	Cohabiting	0	Student
3	62	Ovarian cancer	IV	Married	2	Retired
4	79	Ovarian cancer	I A	Widowed	1	Retired
5	57	Borderline	—	Single	1	Retired
6	66	Ovarian cancer	III C	Married	2	Retired
7	61	Ovarian cancer	III C	Married	3	Civil servant
8	72	Ovarian cancer	III C	Widowed	2	Retired
9	60	Ovarian cancer	I C	Married	2	Employee
10	51	Ovarian cancer	IV	Married	3	Official

^1^International Federation of Gynaecology and Obstetrics.

**Table 2:** Semistructured interview guide.

Preoperative interview (i) The evening before the surgery	Postoperative interview (i) Eight weeks after discharge

Feelings (i) How have you been doing, since surgery was decided? (ii) How are you doing at present? (iii) Do you feel ready to undergo surgery tomorrow?	Experiences of illness and treatment (i) How have you been doing, since your discharge from hospital? (ii) How did you experience your discharge?

Thoughts (i) What are your thoughts about the surgery tomorrow? (ii) What are your thoughts about the days after the operation?	Impact of illness and treatment on everyday life (i) How are you doing at present? (ii) How did you experience your first chemotherapy?^2^

Actions (i) What have you been doing these past days? (ii) Have you made any plans for the next days?	Impact of illness and treatment on future life (i) In what ways has the disease/treatment impacted on your daily life? (ii) What are your thoughts about the near future? (iii) Have you made any plans?

^2^Only women receiving chemotherapy were asked this question.

**Table 3:** Structure analysis of main theme: hope and existential concerns during final diagnostics and first treatment period.

Empirical findings	Meaning condensation	Subthemes
*“I believe that in case of serious illness a certain amount of courage might be helpful—but still, I've seen people with lots of guts succumb to cancer. I've seen that.” *	Courage to face life is important but not a cure	Courage to face life
*“If you're sad sometimes they [the nurses] come and sit with you for a moment—if they can feel there might be something wrong.” *	Care can influence courage to face life	

*“There was this out patient visit [at the department of oncology] where they spoke rather negatively of the effect of chemo. Somehow, this took away my hope. Because… well, I was still alive, but I felt like a really poor life when I left the department.” *	Care influences hope	Hope
*“I certainly haven't given up yet. But I try to be realistic—my notion of becoming a very old lady has been somewhat downplayed, due to this.” *	Notions of the future are being revised	
*“I have a cousin—she's had the same kind of disease. She became a very positive person—I hope the same will happen to me” *	Personal experiences can impact hope	
*“Well, once you've had a cancer disease you've got to live with that. There are no guarantees that it won't ever come back.” *	Life has forever changed	

*“Right from the beginning—if I should cut to the bone—I really think it's about that I haven't ever been afraid of dying.” *	Death becomes a reality	Existential considerations
*“In that sense I still believe that the world can progress. Because, still there are people… who love each other, and who try to understand—I'm part of that” *	Personal hope can be integrated in a universal understanding of life	
*“Of cause it would be terrible [to die right away], but I've lived my life. I've had a good education and children, I've worked for many years, and I've had friends and experiences—a good deal of failures but also some success, right? I've had my life” *	Existential considerations can add meaning to the disease	
*“I believe that someone's holding his hand over me.” *		
